# Tailoring Cell Morphomechanical Perturbations Through Metal Oxide Nanoparticles

**DOI:** 10.1186/s11671-019-2941-y

**Published:** 2019-03-28

**Authors:** Valeria De Matteis, Mariafrancesca Cascione, Chiara Cristina Toma, Paolo Pellegrino, Loris Rizzello, Rosaria Rinaldi

**Affiliations:** 1 Dipartimento di Matematica e Fisica “Ennio De Giorgi”, Università del Salento, Via Arnesano, 73100 Lecce, Italy; 2 Department of Chemistry, University College London, 20 Gordon Street, London, WC1H 0AJ UK; 3 Institute for Bioengineering of Catalonia (IBEC), The Barcelona Institute of Science and Technology, Baldiri Reixac 10-12, 08028 Barcelona, Spain

**Keywords:** Toxicity, Nanoparticles, Cytoskeleton rearrangements, Biomechanics, Young’s Modulus

## Abstract

The nowadays growing use of nanoparticles (NPs) in commercial products does not match a comprehensive understanding of their potential harmfulness. More in vitro investigations are required to address how the physicochemical properties of NPs guide their engulfment within cells and their intracellular trafficking, fate, and toxicity. These nano-bio interactions have not been extensively addressed yet, especially from a mechanical viewpoint. Cell mechanic is a critical indicator of cell health because it regulates processes like cell migration, tissue integrity, and differentiation via cytoskeleton rearrangements. Here, we investigated in vitro the elasticity perturbation of Caco-2 and A549 cell lines, in terms of Young’s modulus modification induced by SiO_2_NP_S_ and TiO_2_NP_S_. TiO_2_NPs demonstrated stronger effects on cell elasticity compared to SiO_2_NPs, as they induced significant morphological and morphometric changes in actin network. TiO_2_NP_S_ increased the elasticity in Caco-2 cells, while opposite effects have been observed on A549 cells. These results demonstrate the existence of a correlation between the alteration of cell elasticity and NPs toxicity that depends, in turn, on the NPs physicochemical properties and the specific cell tested.

## Background

The large use of engineered nanoparticles (ENPs) in commercial products is raising awareness about their potential toxicity to humans and the environment [1]. Many in vitro and in vivo investigations have been conducted so far with the aim to shed light on the molecular mechanisms of toxicity [2, 3]. However, understanding the interactions between nanoparticles (NPs) and living organisms is rather difficult due to the lack of standardized operating procedures, which resulted in the current controversial literature data available [4, 5]. It is established that the adverse effects of NPs strictly depend on their physicochemical properties and on the specific cell or organism tested [6]. For this reason, the characterization of NPs is fundamental to achieve reliable data [7]. Metal oxide NPs are largely widespread in commercial products [8]. Among these, amorphous SiO_2_NPs and crystalline TiO_2_NPs are used in a broad range of industrial fields as additives to drugs and cosmetics and in health care products, printer toners, paints, food packaging, and food additives [9, 10]. Hence, it is likely that these NPs can access living organisms through different routes (ingestion, inhalation, and dermal penetration) [11]. Examples are, but not restricted to, the food products based on TiO_2_NPs (labeled E171 in commercial label) and SiO_2_NPs (E551, E554, E556 in commercial label), which have had a huge growth [12–14]. The current studies on SiO_2_NPs and TiO_2_NPs suggest that they actively interfere with crucial cell mechanisms. For example, they have been proved to stimulate cytokine release (thus promoting inflammation) [15–17] to damage the intestinal microvilli [18, 19], induce ROS production [20], inhibit ATP synthesis [21], and induce genotoxicity [22–26]. Yet, very few studies explored whether these NPs interact with the cell mechanics [27], a topic requiring further investigations. Cell adhesions and cytoskeleton rearrangements are crucial to maintain the cell homeostasis indeed [28]. Any changes in the cytoskeleton architecture can perturb the cellular mechanics and affect cell elasticity and migration dynamics [29]. In this study, we carefully assessed the biomechanical effects of 20 nm SiO_2_NPs and TiO_2_NPs on Caco-2 and A549 cells, which are the best models resembling the tissues exposed to NPs. We preliminarily explored their entry mechanisms, as well as assessed cell viability, membrane damage, and ROS production together with superoxide dismutase (SOD) and malondialdehyde (MDA) activation. Then, we focused on characterizing the changes in cell elasticity (Young’s modulus) upon NPs incubation by atomic force microscopy (AFM). Our results show that NPs may induce a significant reorganization of cortical actin, as confirmed by the changes in Young’s modulus. In particular, a major biocompatibility of SiO_2_NPs against a chronic toxicity of TiO_2_NPs has been observed. Our approach of coupling cytotoxicity investigations with biomechanical characterizations represents a new potential method for standardizing protocols in NP-toxicity assessment.

## Methods

### Synthesis of Amorphous SiO_2_NPs

The ternary W/O microemulsion was prepared at room temperature by mixing water, an organic solvent (Cyclohexane, J.T. Baker), a surfactant (Triton X-100, Sigma-Aldrich) following the methods of Malvindi et al. [25]. Briefly, 880 μL of Triton X-100, 3.75 mL of cyclohexane, 170 mL of water, and 50 μL of TEOS (98%, Sigma-Aldrich) were mixed and stirred for 30 min. Later, 30 μL of NH_4_OH (28.0–30.0%, Sigma-Aldrich) was added to the microemulsion. After 24 h, the suspension was separated by centrifugation (4500 rpm) followed by five washes in ethanol (98%, Sigma-Aldrich), and milliQ water. The nanoparticles were then dispersed in water.

### Synthesis of TiO_2_NPs

TiO_2_NPs were prepared following the sol-gel method described by Leena et al. [30] with some modifications. Briefly, titanium (IV) isopropoxide (TTIP; 99.9% Sigma-Aldrich) was dropped in a solution of ethanol and milliQ water (5:1:1) under stirring in acidic conditions (pH 3). NPs were incubated for 5 h at 30 °C first and then at 430 °C for 3 h to obtain a white nano powder.

### TEM Characterization

Transmission electron microscopy (TEM) characterizations were carried out with a JEOL Jem 1011 microscope, operating at an accelerating voltage of 100 Kv (JEOL USA, Inc.). TEM samples were prepared by dropping a dilute solution of NPs in water on carbon-coated copper grids (Formvar/Carbon 300 Mesh Cu).

### DLS and ζ-Potential Measurements

The average hydrodynamic size and zeta potential of SiO_2_NPs and TiO_2_NPs were determined by dynamic light scattering (DLS) and ζ-potential measurements performed on a Zetasizer Nano-ZS equipped with a 4.0-mW HeNe laser operating at 633 nm and an avalanche photodiode detector (Model ZEN3600, Malvern Instruments Ltd., Malvern, UK). Measurements were made at 25 °C in aqueous solutions and in cell culture medium (DMEM, high glucose, Sigma-Aldrich) supplemented with FBS (Sigma-Aldrich) at 10% and 20% pH 7). Each sample was ran three times, using two independent technical replicates, to obtain the average values of DLS measurements and ζ-potential.

### XRD Characterization

Powder X-ray diffraction (XRD) for crystalline phase analysis of TiO_2_NPs was performed on a Rigaku, diffractometer in Bragg-Brentano reflection geometry using filtered Cu-Ka radiation. The XRD patterns were recorded in the range of 2Q ¼ 20–80 by step scanning, using 2Q increments of 0.02 and a fixed counting time of 2 s/step.

### Cell Culture

Caco-2 (ATCC® HTB-37™) and A549 (ATCC® CCL-185™) were maintained in DMEM with 50 μM glutamine, supplemented with 100 U/mL penicillin and 100 mg/mL streptomycin. The percentage of FBS was 10% for A549 and 20% for Caco-2 cells. Cells were incubated in a humidified controlled atmosphere with a 95 to 5% ratio of air/CO_2_, at 37 °C.

### Determination of the Intracellular Uptake of SiO_2_NPS and TiO_2_ NPs

10^5^ Caco-2 and A549 cells were seeded in 1 mL of medium in a six-well plate. After 24 h of incubation at 37 °C, the medium was replaced with fresh medium containing the SiO_2_NPs and TiO_2_NPs, at concentrations of 15 μg/ml and 45 μg/ml. After 48 h, 72 h, and 96 h of incubation at 37 °C, DMEM was removed, and the cells washed four times with PBS (pH 7.4), to remove NPs that could be bound to the cellular membrane. Cells were trypsinized and counted using automatic cell counting chamber. Three hundred sixty thousand cells were suspended in 200 μL of milliQ and treated with HCl/HNO_3_ 3:1 (*v*/*v*) and diluted to 5 mL: the resulting solution was analyzed to evaluate Si and Ti content. Elemental analysis was carried out by inductively coupled plasma atomic emission spectroscopy (ICP-AES) with a Varian Vista AX spectrometer.

### WST-8 Assay

Caco-2 and A549 cells were seeded in 96-well microplates at concentration of 5 × 10^3^ cells/well after 24 h of stabilization. NP stock solutions (SiO_2_NPs and TiO_2_NPs) were added to the cell media at 15 μg/ml and 45 μg/ml. Cells were incubated for 24 h, 48 h, 72 h, and 96 h. At the endpoint, cell viability was determined using a standard WST-8 assay (Sigma-Aldrich). Assays were performed following the procedure previously described in De Matteis et al. [31]. Data were expressed as mean ± SD.

### LDH Assay

Caco-2 and A549 cells were treated with SiO_2_NPs and TiO_2_NPs following the procedure reported for the WST-8 assay. The lactate dehydrogenase (LDH) assay was performed on microplates by applying the CytoTox-ONE Homogeneous Membrane Integrity Assay reagent (Promega), following the manufacturer’s instructions. The culture medium was collected, and the level of LDH was measured by reading absorbance at 490 nm using a Bio-Rad microplate spectrophotometer. Data were expressed as mean ± SD.

### DCF-DA Assay

Caco-2 and A549 cells were seeded in 96-well microplates and treated with SiO_2_NPs and TiO_2_NPs at a final concentrations of 15 μg/ml and 45 μg/ml. After 24 h, 48 h, 72 h, and 96 h of cell–NP interaction, the DCF-DA (Sigma) assay was performed onto microplates following the procedure reported by De Matteis et al. [32] Data were expressed as mean ± SD.

### SOD Assay

Caco-2 and A549 (incubated with 15 μg/ml, 45 μg/ml for 24 h, 48 h, 72 h, and 96 h) cell extracts were prepared according to the protocol described in [33]. The assay was performed on microplates by applying a SOD assay (Cayman Chemical Company, Michigan, OH, USA) that measures all three types of SOD (Cu/ZnSOD, MnSOD, and FeSOD). The assay used a tetrazolium salt for detection of superoxide radicals generated by xanthine oxidase and hypoxanthine. One unit of SOD is defined as the amount of enzyme needed to exhibit 50% dismutation of the superoxide radical. The SOD activity was measured by reading absorbance at 440–460 nm using a Bio-Rad microplate spectrophotometer.

### MDA Assay

Caco-2 and A549 (incubated with 15 μg/ml, 45 μg/ml for 24 h, 48 h, 72 h, and 96 h) cell extracts were prepared according to the previously described procedures [33]. The assay was performed on microplates by applying Lipid Peroxidation (MDA) Assay kit (Abcam): the MDA in the sample reacted with thiobarbituric acid (TBA) to generate a MDA-TBA adduct. This route involved the spectrophotometric measurement of the red color produced during the formation of MDA-TBA adduct, which can be quantified (in terms of nmol/mg protein) by reading absorbance at 532 nm using a Bio-Rad microplate spectrophotometer.

### CLSM Analysis

Cells were seeded in 24-well plate at concentration of 10^5^ cells/well and successively incubated with SiO_2_NPs and TiO_2_NPs at concentration of 15 μg/ml and 45 μg/ml for 24 h, 48 h, 72 h, and 96 h. After treatment, for each time point, the medium containing nanoparticles was removed and the cells were washed three times with PBS, fixed with 0.25% glutaraldehyde (*v*/*v* in PBS, Sigma-Aldrich) for 20 min, and finally permeabilized with 0.1% Triton (*v*/*v* in PBS, Sigma-Aldrich) for 5 min For the actin staining, Phalloidin–ATTO 488 (Sigma-Aldrich) was used at concentration of 1 μg/ml for 30 min. Nuclei were marked by means of DAPI (Sigma-Aldrich) at concentration of 1 μg/ml for 7 min. Laser scanning confocal microscopy was performed on a Zeiss LSM700 (Zeiss) confocal microscope equipped with an Axio Observer Z1 (Zeiss) inverted microscope using × 100, 1.46 numerical aperture oil immersion lens for imaging. Confocal data files were processed using ZEN2010 software (Zeiss), and morphometric quantifications (coherency and integrated density of F-actin) were performed on 15 cells, using the ImageJ 1.47 analysis software. OrientationJ plugin was used to quantify the coherency parameter by choosing a specific sequence of ROIs in confocal acquisitions, based on the measure of the structure tensors in a local neighborhood. At the same time, the software calculated the value of orientation and coherency that represented the degree to which the actin fibers were oriented: more disordered fibers have values near 0, whereas perfectly aligned ones show coherency value of about 1 [34]. Integrated density was also calculated by the sum of the pixels values in the ROIs on confocal acquisitions in order to quantify the amount of actin fibers in cells.

### AFM Analysis

Caco-2 and A549 cells were seeded in plastic Petri dishes (Corning) at a concentration of 10^5^ cell/well and grown until a 70–80% of confluence. Cells were then treated with 45 μg/ml of a TiO_2_NP_S_ and SiO_2_NPs in DMEM for 72 h. Successively, NPs were removed and the cells washed with PBS. Cells were fixed using glutaraldehyde 0.25% for 20 min, followed by washing with PBS. The measurements were conducted by an advanced scanning probe microscope (Bioscope Catalyst, Bruker Inc., USA) mounted on an inverted optical microscope (Zeiss Observer Z1, Zeiss GERMANY). The whole system is placed on a base that acts as an insulator with respect to the environmental mechanical vibrations. AFM experiments were performed in force–volume mode by using V-shaped Bruker’s Sharp Microlever (MSNL, tip C): a high-sensitivity silicon nitride cantilever with nominal spring constant of 0.01 N/m. This value was accurately estimated by thermal tune method [35] earlier than carry out AFM acquisitions. Parameters used were as follows: scan area 50 μm, ramp rate 3 Hz, FV scan rate 0.03 Hz, trigger threshold 100 nm, number of sample 128, sample per line 64, and lines 64. The Young’s modulus (E) was determined on 20 cells, from which 25 force–distance curves were extracted in correspondence of nuclear area and 25 curves in cytoplasmic region. The approach data (from contact point to maximum force value) set derived from the extracted curves was fitted with a modified Sneddon model:

$$ -{k}_{\mathrm{c}}{\delta}_{\mathrm{c}}=\frac{2 Etg\alpha}{\pi \left(1-{\nu}^2\right)}{\left(z-{\delta}_{\mathrm{c}}\right)}^2 $$

where *z* and δ*c* were the experimental loading data (height and cantilever deflection, respectively), *α* is half-angle of tip, *k*_c_ was the elastic constant value of cantilever, and *ν* is the Poisson ratio (assumed to be 0.5 for biological sample). In the fit algorithm, the contact point was treated as fit variable and the adhesion forces were taken into account were acquired on 20 cells.

### Statistical Analysis

Data were expressed as mean value and associated standard deviation. Differences between different mean values were considered statistically significant performing the Student *t* test with a *p* value ˂ 0.05 (< 0.05*, < 0.01**, and < 0.005***).

## Results

### Characterization of SiO_2_NPs and TiO_2_NPs

**Fig. 1:**
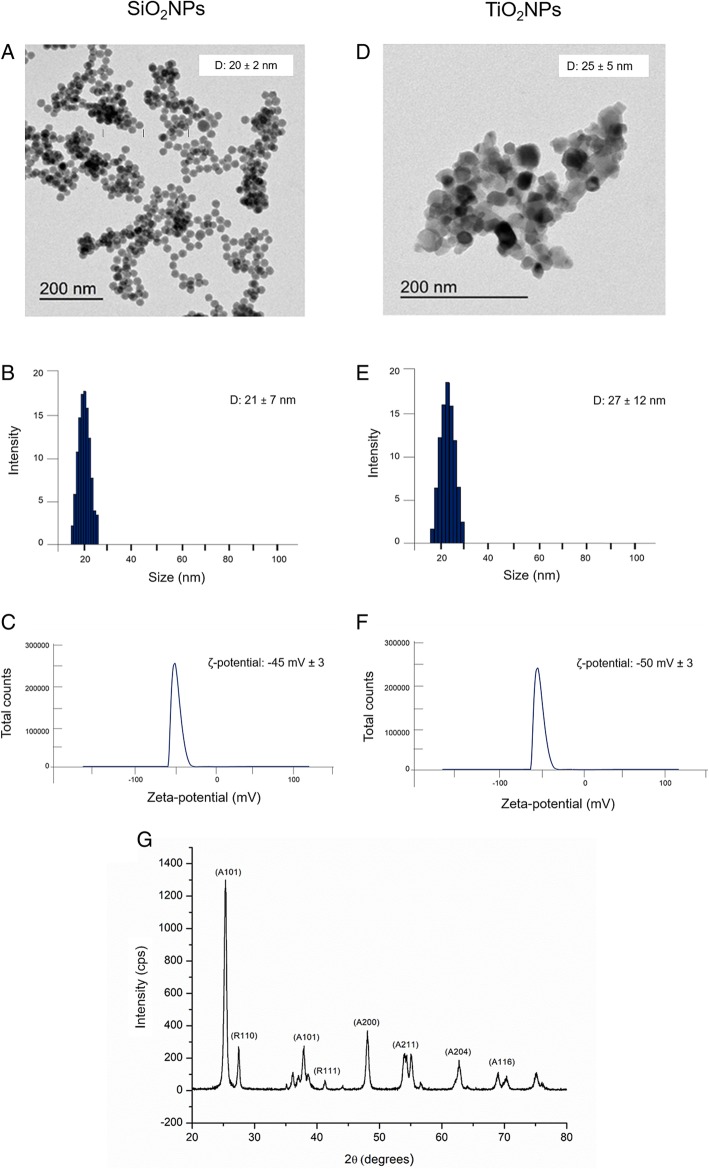
Characterizations of SiO_2_NPs and TiO_2_NPs in water. **a**–**d** Representative TEM images. **b**–**e** Dynamic light scattering (DLS) and **c**–**f** ζ-potential measurements. **g** X-ray diffraction analysis (XRD) pattern of TiO_2_NPs

SiO_2_NPs and TiO_2_NPs have been synthetized with different and reproducible synthetic routes in order to obtain NPs having a narrow and controlled size distribution (see “Methods” section). Then, NPs were deeply characterized by means of TEM, DLS, ζ-potential, and XRD, both in water and in the cell culture media (DMEM) with different concentrations of protein source (FBS). This is crucial, as the media proteins can cover the NPs surface, thus changing their physicochemical properties and, hence, the biological effects [36]. TEM analyses showed that SiO_2_NPs are spherical in shape, with an average diameter of 20 ± 2 nm (Fig. 1a). TiO_2_NPs have a similar size (25 ± 5 nm), but different morphology (Fig. 1). DLS measurements carried out in water at 96 h confirmed a hydrodynamic radius of 21 ± 7 nm and 27 ± 12 nm for SiO_2_NPs and TiO_2_NPs, respectively (Fig. 1b and Fig. 1e). As expected, these data are in good agreement with the TEM observations. ζ-Potential analyses also confirmed surface charge values in water of − 45 ± 3 mV for SiO_2_NPs and of − 50 ± 3 mV for TiO_2_NPs (Fig. 1c, f). As expected, the physicochemical properties of the NPs changed upon inoculation within the cell culture media. DLS confirmed a significant increase in NP size especially in the presence of DMEM supplemented with 20% of FBS (Table 1). In particular, SiO_2_NPs showed a size of 29 ± 9 nm, while TiO_2_NPs increased up to 41 ± 14 nm after 96 h. The enlargement of the DLS peak observed in the DMEM measurements (with or without FBS) is a sign of NPs agglomeration, which can be promoted by the ionic strength of the medium (data not shown). Also, the ζ-potential measurements demonstrated that the surface charge of both NPs shifted to more negative values**.** This large time-dependent phenomenon was due to the quite stable protein corona formation [37, 38] induced by the presence of serum proteins in cell culture media that were adsorbed on NPs’ surface: the size and the charge of NPs change as a function of the FBS concentration.

**Table 1:** Characterization of SiO_2_NPs and TiO_2_NPs in DMEM supplemented with 10% and 20% of FBS

SiO_2_NPS in DMEM 10% FBS	Values	SiO_2_NPS in DMEM 20% FBS	Values
Dynamic light scattering (DLS)	25 ± 5 nm	Dynamic light scattering (DLS)	29 ± 9 nm
Zeta potential	− 55 ± 6 mV	Zeta potential	− 57 ± 6 mV
TiO_2_NPS in DMEM 10% FBS	Values	TiO_2_NPS in DMEM 20% FBS	Values
Dynamic light scattering (DLS)	33 ± 10 nm	Dynamic light scattering (DLS)	41 ± 14 nm
Zeta potential	− 57 ± 5 mV	Zeta potential	− 59 ± 6 mV

The XRD pattern of TiO_2_NP_S_, calcinated at 430 °C, showed a mixture of anatase and rutile crystalline phases (Fig. 1g**)**. The dominant peaks at 2θ = 25.4° (101), 48.1° (200), 54.1° (211), 62.4° (204), and 68.8° (116) were distinctive of the anatase phase matching well to the standard JCPDS data (card no: 21–1272). Rutile phase was represented with diffraction peaks at 27.5° (110), 36.2° (101), and 41.2° (111).

### Uptake of NPs in Caco-2 and A549 Cells

**Fig. 2:**
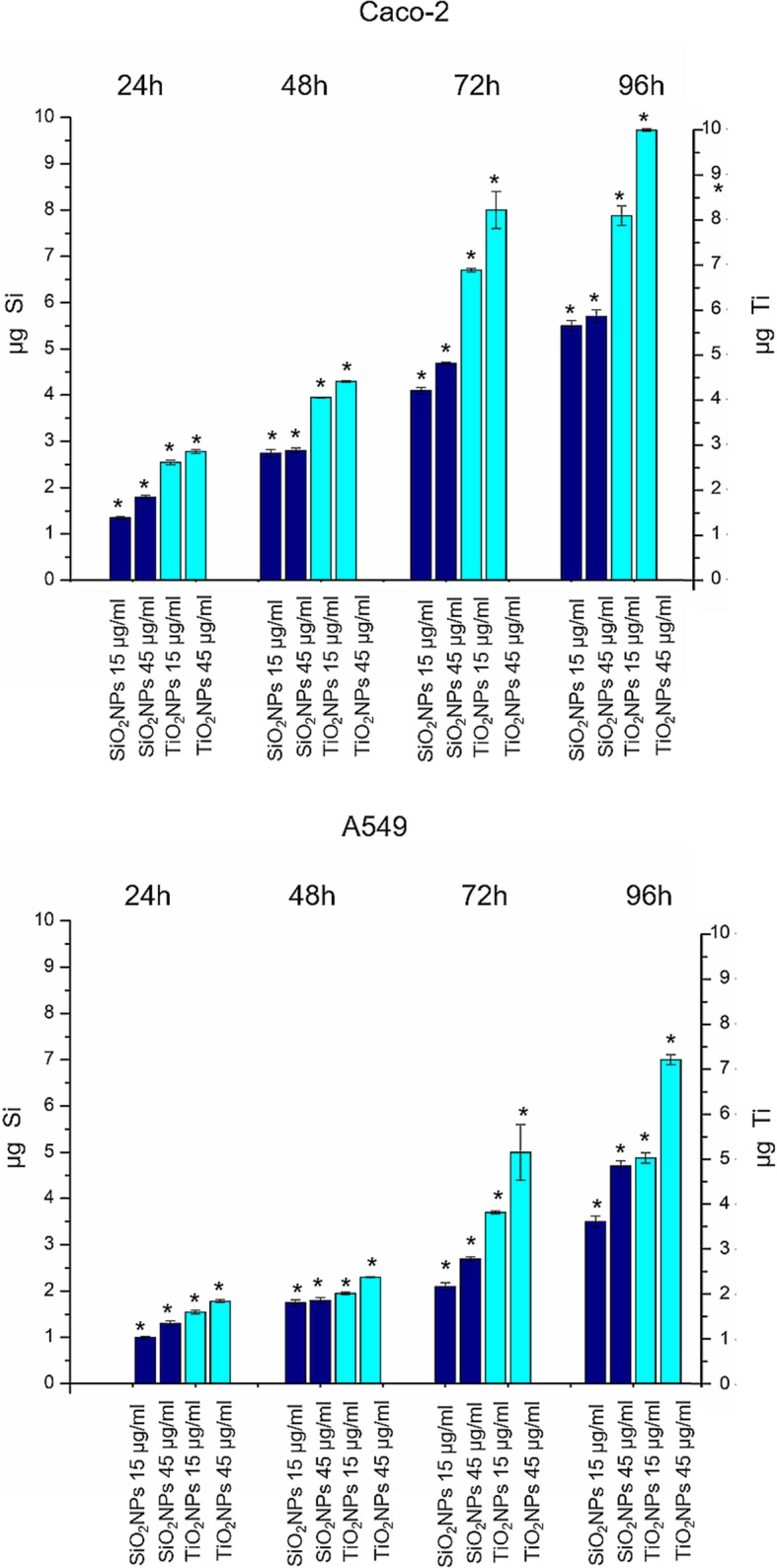
TiO_2_NPs and SiO_2_NPs accumulation in Caco-2 and A549 cell lines exposed to 15 μg/ml and 45 μg/ml of TiO_2_NPs and SiO_2_NPs for 24 h, 48 h, 72 h, and 96 h. Cells were then harvested, live cells were counted, and Ti and Si content was measured in 360,000 cells (μg Ti and μg Si). Data reported as mean ± SD from three independent experiments; statistical significance of exposed cells vs. control cells for *p* value < 0.05 (< 0.05*, < 0.01**, and < 0.005***)

In order to quantify the amount of SiO_2_NPs and TiO_2_NP_S_ taken-up by cells, we performed ICP-AES elemental analysis over lysed cell as preliminary investigation. Cells were treated with 15 μg/ml and 45 μg/ml of NPs. The experimental data confirmed the presence of SiO_2_NPs and TiO_2_NPs in both cell lines, with a time-dependent internalization efficiency (Fig. 2a). TiO_2_NPs showed a larger uptake with the respect to SiO_2_NPs. This was particularly evident in the Caco-2, where the Ti content reached intracellular concentrations of 8.2 ± 0.4 μg and 9.7 ± 0.031 μg after 72 h and 96 h, respectively**.** The amount of Ti detected in the A549 was lower, as we found 5 ± 0.599 μg after 72 h and 7.12 ± 0.11 μg after 96 h of incubation time. SiO_2_NPs were less taken-up by cells compared with TiO_2_NPs, even if the internalization was more pronounced in Caco-2. Also in this case, in fact, the amount of internalized SiO_2_NPs in Caco-2 cells was 4.69 ± 0.031 μg after 72 h and 5.78 ± 0.045 μg after 96 h of incubation. The values decreased in A549, where we quantified 2.58 ± 0.045 μg after 72 h and 4.7 ± 0.04 μg after 96 h.

### Effects of NPs on CaCo-2 and A549: Cells Viability, Membrane Damage and ROS Production

**Fig. 3:**
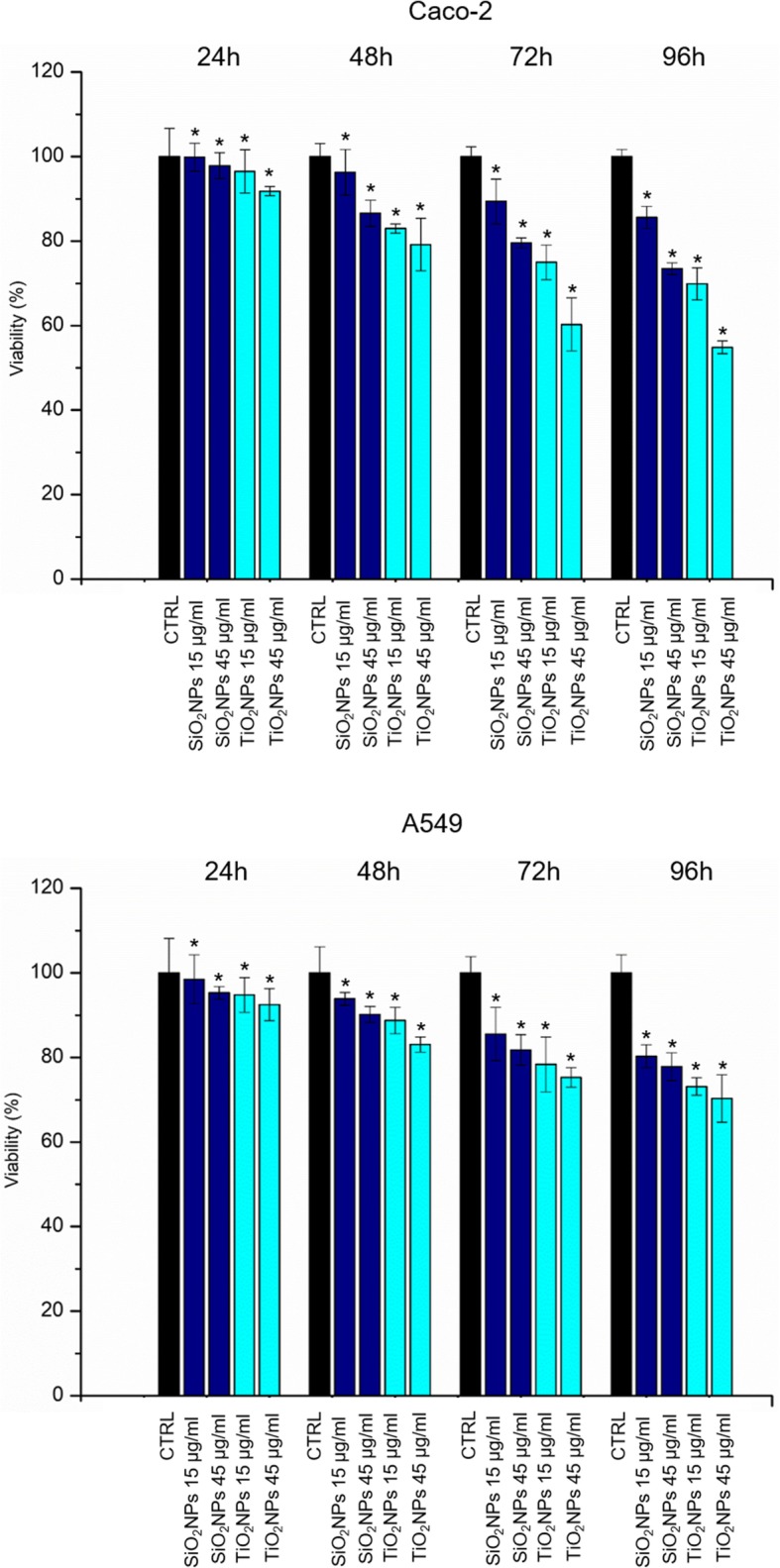
Viability assay (WST-8) of Caco-2 (**a**) and A549 (**b**) cells after 24 h, 48 h, 72 h, and 96 h of exposure to two doses (15 μg/ml and 45 μg/ml) of TiO_2_NPs and SiO_2_NPs. Viability of NP-treated cells was normalized to non-treated control cells. As positive control (P), cells were incubated with 5% DMSO (data not shown). Data reported as mean ± SD from three independent experiments are considered statistically significant compared with control (*n* = 8) for *p* value < 0.05 (< 0.05*, < 0.01**, and < 0.005***)

**Fig. 4:**
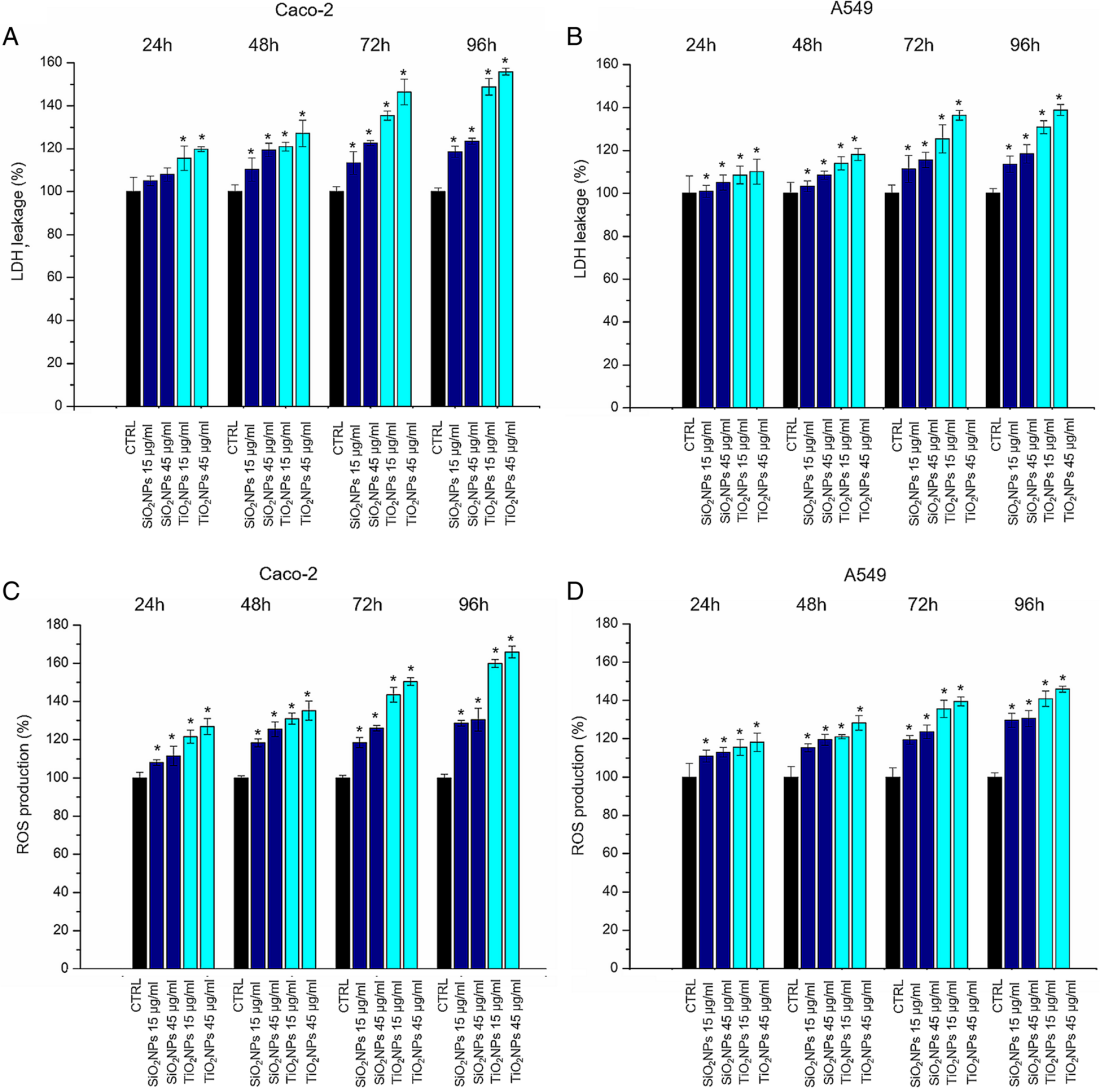
LDH (**a**–**b**) and ROS (**c**–**d**) assays on Caco-2 and A549 cells. Cells were incubated with 15 μg/ml and 45 μg/ml of TiO_2_NPs and SiO_2_NPs for 24 h, 48 h, 72 h, and 96 h. Percent of LDH leakage of nanoparticle-treated cells are expressed relative to non-treated control cells. Positive controls (P) consisted in the treatment of cells with 0.9% Triton X-100 showing ca. 500% LDH increase (data not shown). ROS levels were recorded exposing Caco-2 and A549 cells with 15 μg/ml and 45 μg/ml of TiO_2_NPs and SiO_2_NPs for 24 h, 48 h, 72 h, and 96 h incubated with 100 μM DCFH-DA. Cell fluorescence was measured. As a positive control (P), cells were incubated with 500 μM H_2_O_2_ showing a ca. 300% DCFH-DA increase (not show). Data reported as mean ± SD from three independent experiments are considered statistically significant compared with control (*n* = 8) for *p* value < 0.05 (< 0.05*, < 0.01**, and < .005***)

The Caco-2 and A549 cell viability was evaluated with the WST-8 assay. The treatment with SiO_2_NPs and TiO_2_NPs induced a slight dose-dependent reduction of viability in both the cell lines tested (Fig. 3). TiO_2_NPs induced an enhanced cytotoxicity with respect to SiO_2_NPs, and the cell viability of CaCo-2 cells was more affected than the A549, upon treatment with TiO_2_NPs. In particular, we observed a reduction of viability of about 40% in Caco-2 treated with 45 μg/ml of TiO_2_NPs for 72 h. This reduction further dropped down up to 50% after 96 h, whereas, in A549 cell lines, TiO_2_NPs induced a reduction of 30% of viability only after 96 h of treatment. The LDH release and ROS production were evaluated in Caco-2 and A549 cells upon the exposure to TiO_2_NPs and SiO_2_NPs. As shown in Fig. 4a, b, NPs induced cell membrane poration (and LDH release indeed) in close agreement with the viability results. The effect was more evident in Caco-2 with respect to A549 especially upon TiO_2_NP treatment, at the highest time points (72 and 96 h). The LDH release percentage reached an increase of about 160% with respect to the untreated (control) cells, after 96 h of exposure. The ROS generation has been wildly studied because it is one of the major effects induced by NPs [39]. This phenomenon interferes in biological antioxidant defense response [40], even though it is important mentioning that the real action mechanism is still under investigations. The potential NP-induced oxidative stress was estimated by DCFH-DA assay. As expected, the interaction between NPs and cells stimulated the generation of ROS, in a dose-dependent manner with a strong effect in Caco-2 upon TiO_2_NP treatment (Fig. 4c, d). The percentage of release reached values of 165% with respect to the control cells, at the highest concentration tested.

### Effects Induced by NPs on Antioxidants Activity and Lipid Peroxidation in Caco-2 and A549 Cells

**Fig. 5:**
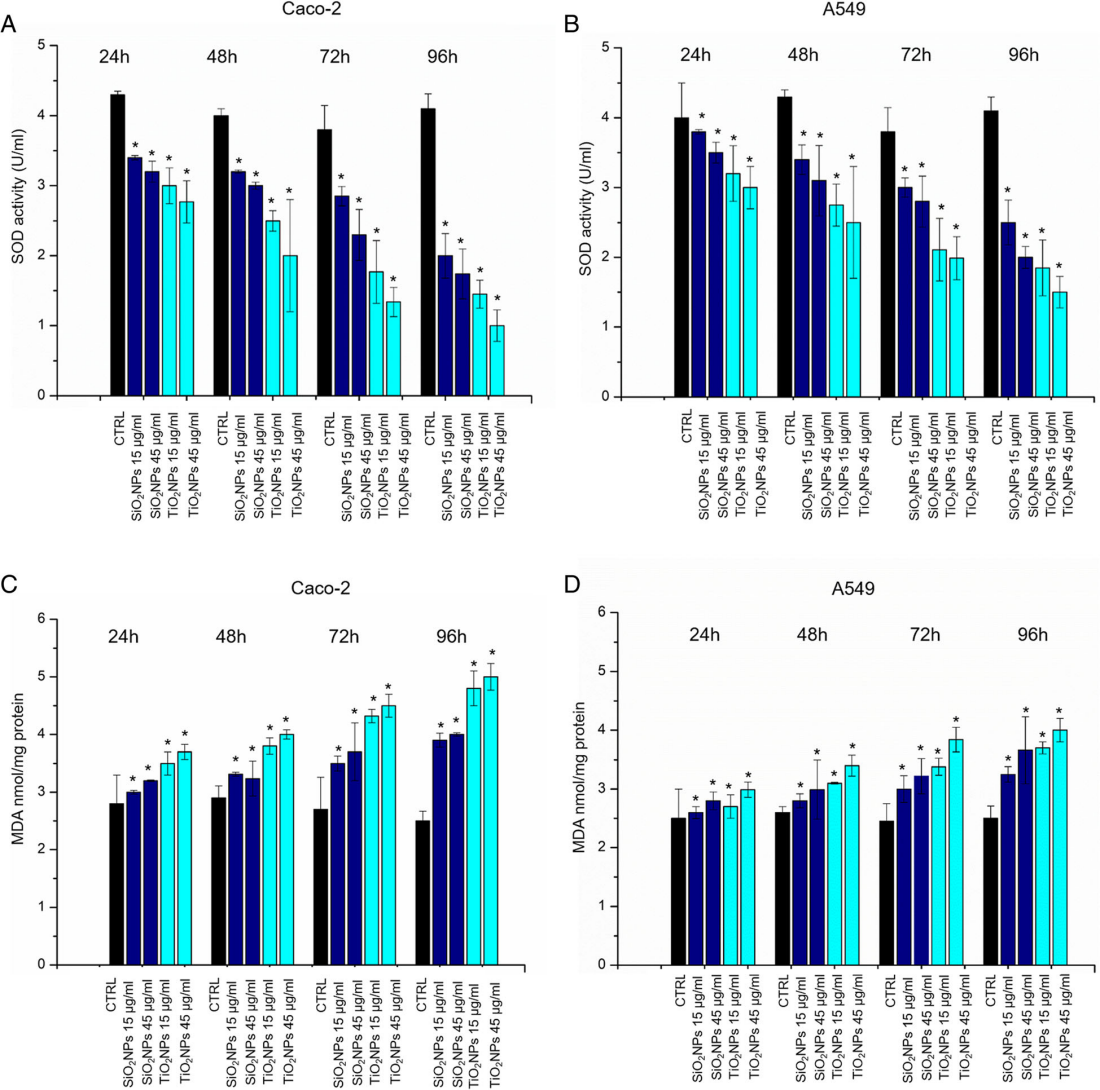
**a**–**d** SOD and MDA assays on Caco-2 and A549 cells. Cells were incubated with 15 μg/ml and 45 μg/ml of TiO_2_NPs and SiO_2_NPs for 24 h, 48 h, 72 h, 96 h. The SOD assay used a tetrazolium salt for detection of superoxide radicals generated by xanthine oxidase and hypoxanthine. The standard curve was used as a positive control (data not shown). The MDA levels was detected by the quantification of MDA-TBA adduct (OD = 532 nm). Data reported as mean ± SD from three independent experiments are considered statistically significant compared with control (*n* = 8) for *p* value < 0.05 (< 0.05*, < 0.01**, and < 0.005***)

SOD enzyme is involved in antioxidant defense system after oxidative stress induction. This enzyme catalyzes the dismutation of highly reactive superoxide (O_2_^•−^) anion into peroxides H_2_O_2_ [41]. We observed a dose-dependent reduction in SOD enzyme activity in both Caco-2 and A549 after incubation with SiO_2_NPs and TiO_2_NPs (15 μg/ml, 45 μg/ml) at different time points (from 24 to 96 h) (Fig. 5a, b). In close agreement with the cytotoxicity assessments, the effect was more evident in the Caco-2 upon TiO_2_NP exposure. For example, the SOD activity levels were reduced from 4.1 ± 0.2 U/ml in the control to 1.03 ± 0.325 U/ml in Caco-2 cells exposed to 45 μg/ml of TiO_2_NPs, after 96 h. The exposure to the same concentration of SiO_2_NPs reduced the SOD activity to 1.45 ± 0.12 U/ml. The MDA-based assay was used to check potential ROS-mediated lipid peroxidation, which is in turn another way to check over cell oxidative stress. [42] The cellular levels of MDA grew after exposure to the two types of NPs for both Caco-2 and A549 (Fig. 5c, d). As expected, the increased MDA levels were proportional to the concentration and exposure time.

### Morphomechanical Effects Induced by NPs

**Fig. 6:**
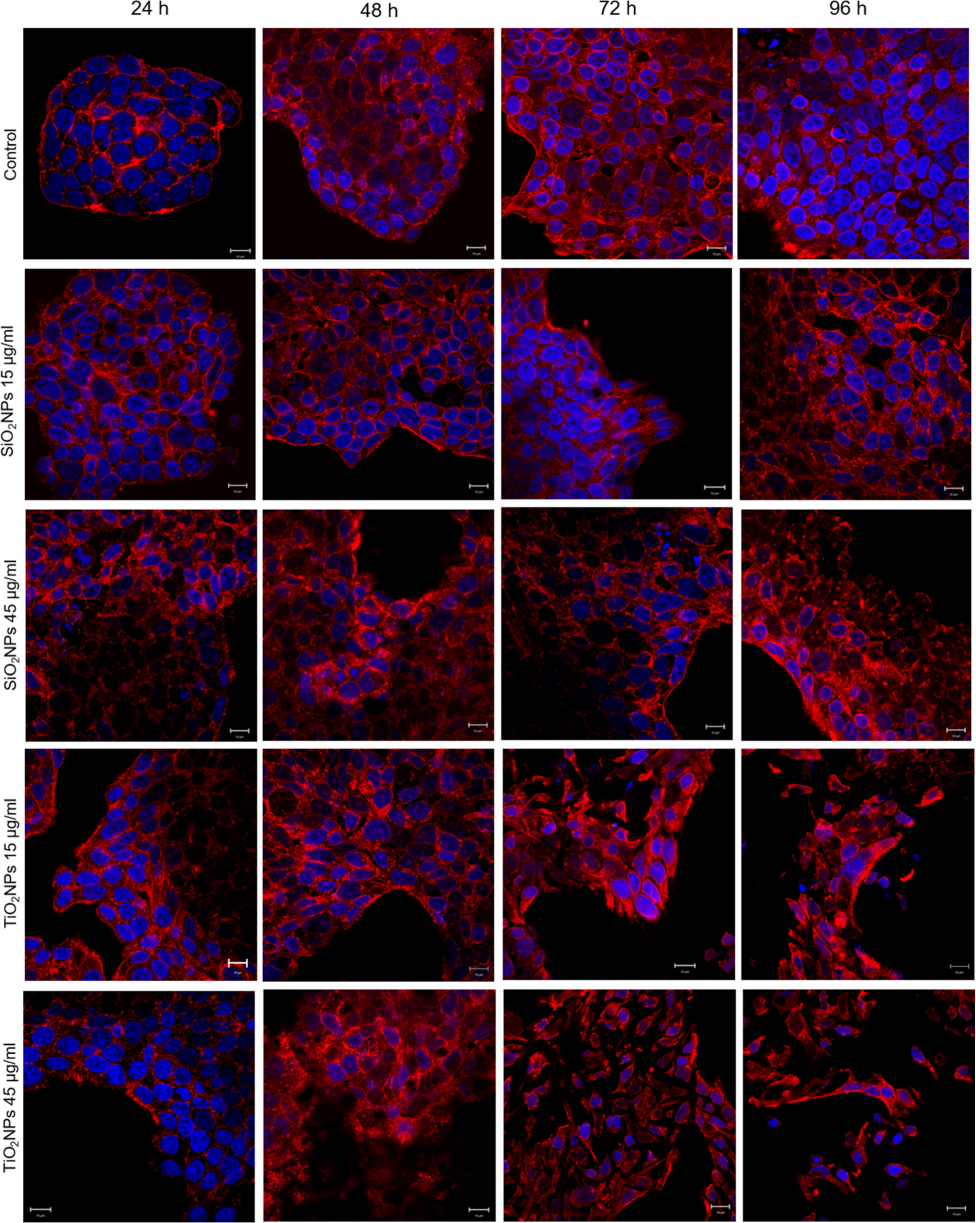
Effects of SiO_2_NPS and TiO_2_NPs on actin network of Caco-2 cells. Caco2 were treated with 15 μg/ml and 45 μg/ml of NPs for 24 h, 48 h, 72 h, and 96 h; cells were fixed and then stained with Phalloidin–ATTO 488 and DAPI. The 2D images of cortical actin were acquired by a Zeiss LSM700 (Zeiss) confocal microscope equipped with an Axio Observer Z1 (Zeiss) inverted microscope using a × 100, 1.46 numerical aperture oil immersion lens. All data were processed by ZEN software (Zeiss)

**Fig. 7:**
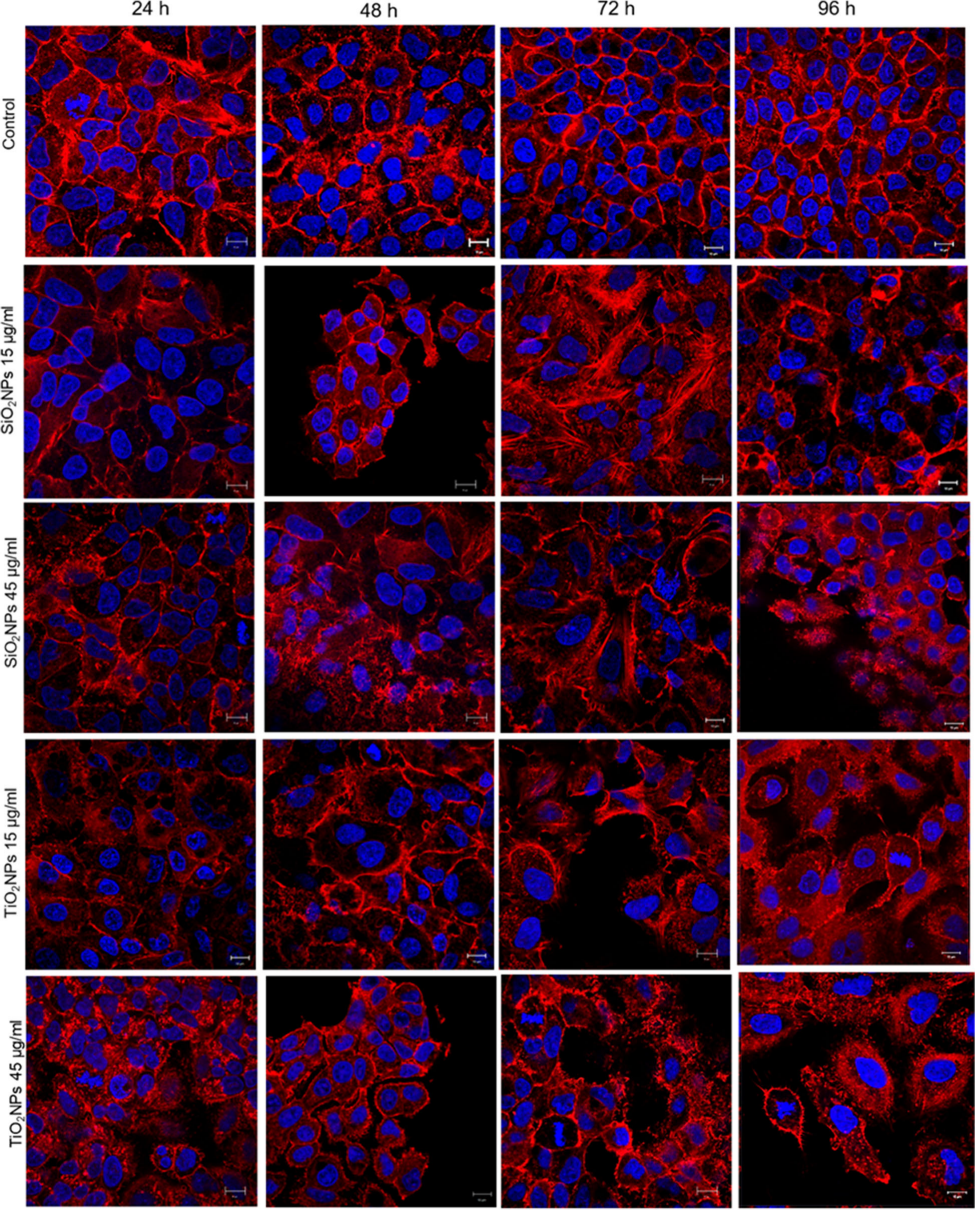
Effect of SiO_2_NPS and TiO_2_NPs on actin network on A549 cells. A549 were treated with 15 μg/ml and 45 μg/ml of NPs for 24 h, 48 h, 72 h, and 96 h; successively they were fixed and stained with Phalloidin–ATTO 488 and DAPI. The 2D images of cortical actin were acquired by a Zeiss LSM700 (Zeiss) confocal microscope equipped with an Axio Observer Z1 (Zeiss) inverted microscope using a × 100, 1.46 numerical aperture oil immersion lens. All data were processed by ZEN software (Zeiss)

**Fig. 8:**
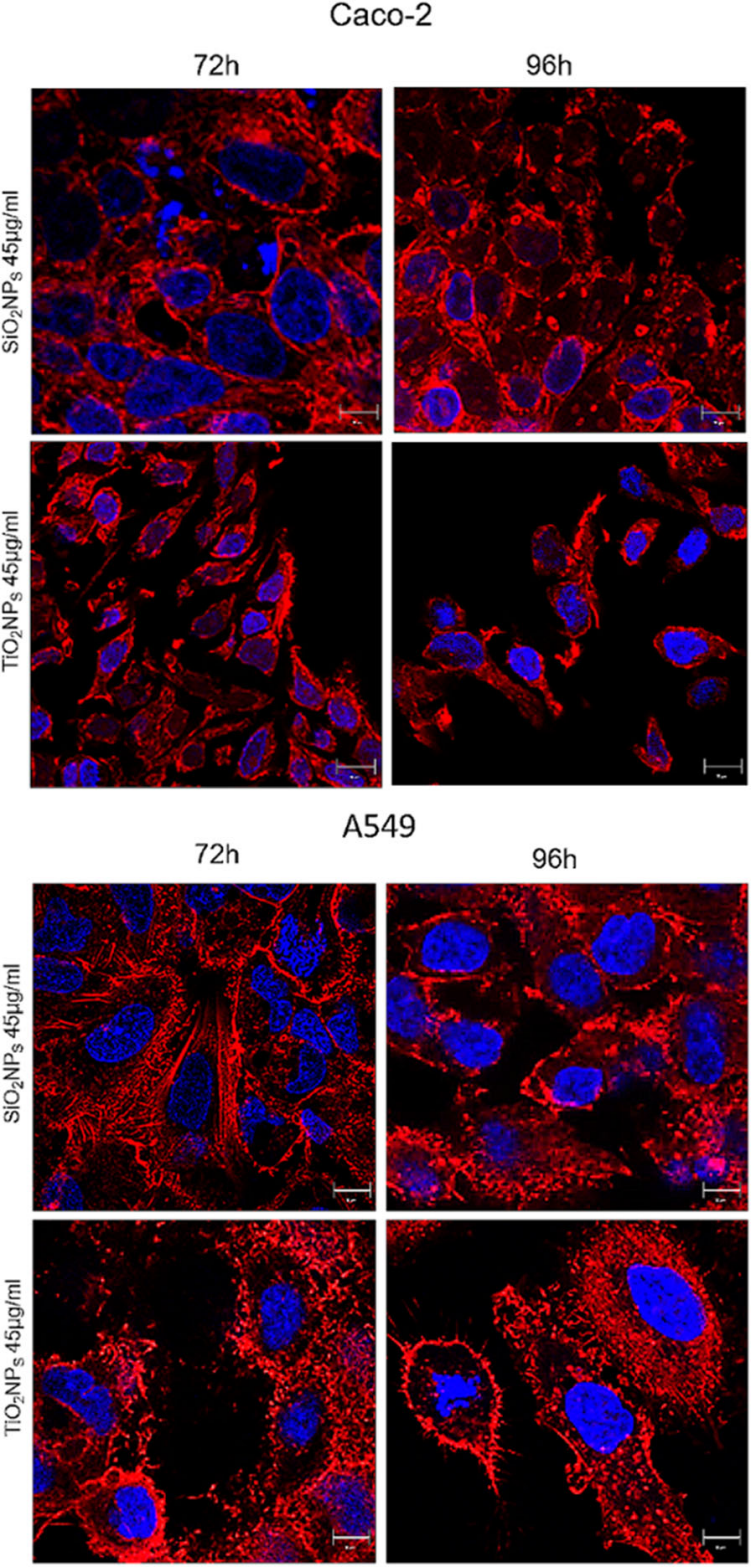
Local enlargement of confocal acquisitions acquired in Figs. 6 and 7 showed (more in details) the effect of SiO_2_NPS and TiO_2_NPs on actin network of Caco-2 and A549 cells after the exposure of 45 μg/ml of NPs for 72 h and 96 h

**Fig. 9:**
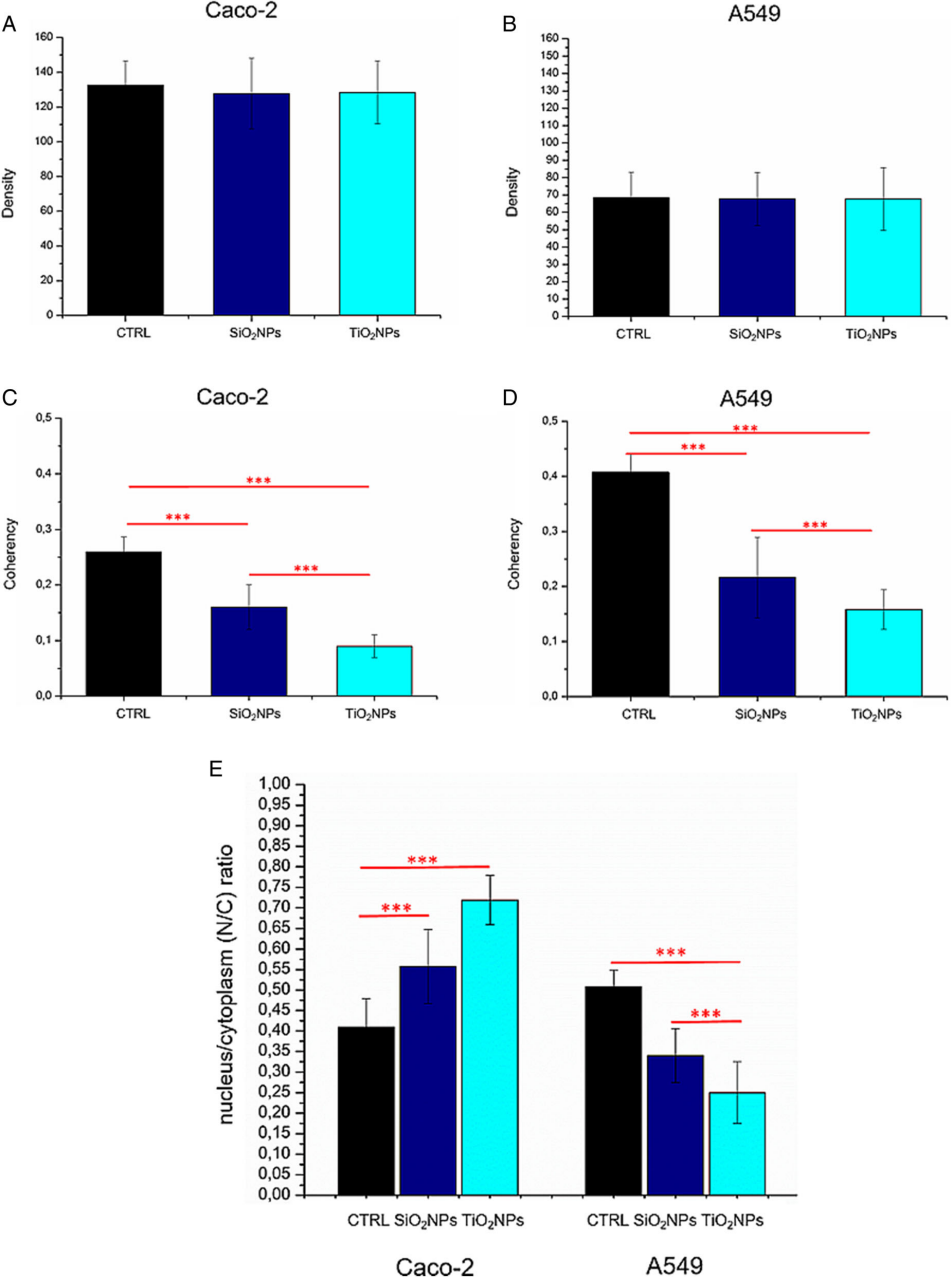
Integrated density (**a**, **b**) and coherency (**c**, **d**) for Caco-2 and A549 cells treated with 45 μg/ml of SiO_2_NPs and TiO_2_NPs after 72 h. The integrated density and coherency values were expressed as a mean value and relative SD, calculated from confocal acquisitions by ImageJ (calculation on 15 cells). The mean values and their standard deviations were reported in the histograms. Results were statistically significant for *p* < 0.05 (< 0.05*, < 0.01**, and < 0.005***). **e** Analyses of nucleus/cytoplasm ratio on Caco-2 and A549 after exposure to 45 μg/ml of SiO_2_NPs and TiO_2_NPs for 72 h. The ratio was calculated on 15 cells by ImageJ. The mean values and the SD were reported in the histogram. Results were statistically significant for *p* < 0.05 (< 0.05*, < 0.01**, and < 0.005***)

**Fig. 10:**
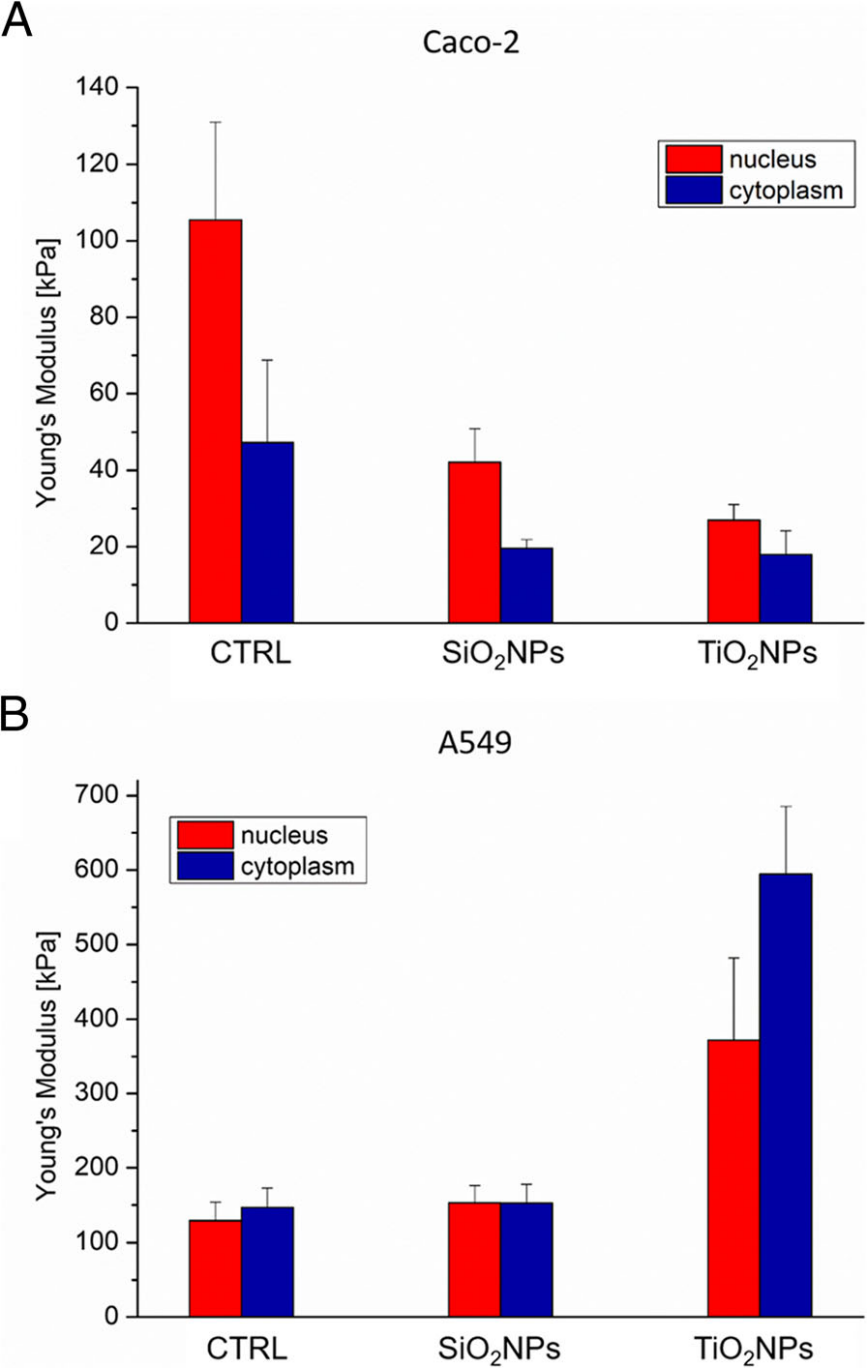
Young’s modulus values expressed in kPa, calculated from the nuclear region and the cytoskeletal area of Caco-2 (**a**) and A549 (**b**) cell lines after a treatment to 45 μg/ml of SiO_2_NPs and TiO_2_NPs for 72 h

Confocal microscopy analyses of Caco-2 and A549 incubated with 15 μg/ml and 45 μg/ml of SiO_2_NPs and TiO_2_NPs for 24 h, 48 h, 72 h, and 96 h are reported in Figs. 6 and 7. Control Caco-2 cells exhibited a morphology similar to intestinal enterocytes with tight junctions and brush border at the apical side [43]. Upon treatment with NPs, cells’ tight junctions collapsed and the cells’ pattern resulted to be isolated, with an elongated shape. These effects were more evident when cells were treated with TiO_2_NPs at 45 μg/ml for 72 h of incubation time, showing relevant alterations of the actin patterns, as well as changes in the cell morphology. The untreated A549 cells displayed a pebble-like shape and functional cell–cell adhesions [44], while the treatment with NPs decreased the cell–cell contacts and modify cell morphology towards more elongated shapes. Figure 8 shows a zoomed-in confocal figure, which enable detecting changes in the actin distribution. The altered organization of actin network after NP exposure (72 h of 45 μg/ml of SiO_2_NPs and TiO_2_NPs) was quantitatively analyzed by fluorescence density and coherency using ImageJ software (Fig. 9). We specifically opted for these two parameters because the integrated density allowed us to quantify the amount of actin, while coherency gave us information on the degree of fiber orientation compared to the surroundings [45]. Untreated Caco-2 cells had a density value of 129.4 ± 16, and this remained unaffected upon NP treatments; the values were 127.7 ± 20 and 128.5 ± 18 after exposure to SiO_2_NPs and TiO_2_NPs, respectively, Fig. 9a). Similarly, also the density of actin-stained network remained the same in A549 before and after treatment (68.4 ± 14, 67.9 ± 15, and 67.7 ± 18 for negative control, SiO_2_NPs, and TiO_2_NPs, respectively, Fig. 9b). Although NP treatments did not induce alteration in the amount of actin, the coherency analyses suggested dissimilar actin spatial reorganization. In Caco-2, the coherency values of treated cells for SiO_2_NP (0.16 ± 0.04) and for TiO_2_NP (0.09 ± 0.02) treatment decreased with respect to that of the control (0.26 ± 0.03) (Fig. 9c). Even the A549 cells underwent a decrease of coherency after interacting with SiO_2_NPs and TiO_2_NPs (0.2 ± 0.07 and 0.158 ± 0.04) compared to the control cells (0.4 ± 0.03) (Fig. 9d). Hence, NPs induced a significant reorganization of actin network, which showed an actin isotropic orientation, but they did not change the overall quantity of actin expressed. In addition to cytoskeletal rearrangements, we also analyzed the area described by the nucleus/cytoplasm ratio. Values of N/C ratio were calculated as the ratio between nuclear area and the whole cellular area (measured performed on 15 cells). We observed opposite values following the treatment with 45 μg/ml of NPs for 72 h with significant statistical validity. In particular, the ratio was (0.40 ± 1.7) in untreated Caco-2 cells, and this increased up to 0.554 ± 0.09 and 0.62 ± 0.12 after SiO_2_NP and TiO_2_NP exposure (Fig. 9e). The trend was different in A549. The nuclear/cytoplasm ratio dropped down upon exposure to NPs from values of 0.550 ± 0.04 for control cells to 0.334 ± 0.06 for SiO_2_NPs and 0.225 ± 0.09 for TiO_2_NPs. After the morphological investigations, we analyzed the elastic properties of cells after exposing them to 45 μg/ml of SiO_2_NPs and TiO_2_NPs for 72 h by AFM, in force–volume mode. We measured the different elasticity expressed by Young’s modulus values in the nuclear and cytoplasmic region. Caco-2 cells displayed Young’s modulus value of 105 ± 25 kPa for nuclear region and 47 ± 21 kPa for the cytoplasm. After SiO_2_NP treatment, we observed a reduction of value to 42 ± 8 kPa for the nucleus and 19.59 ± 2 kPa for the cytoplasm. The effects were more evident after treatment with TiO_2_NPs: the Young modulus for the nucleus was 27 ± 4 kPa and 18 ± 4 kPa for the cytoplasm (Fig. 10a). We found an opposite outcome concerning the elastic properties of A549 cells. In this case, Young’s modulus was 129 ± 24 kPa for the nuclear region and 147 ± 26 kPa for the cytoplasmic area. After SiO_2_NP treatment, the values of elasticity increased to 152 ± 23 kPa for nucleus and 152 ± 25 kPa for cytoplasm. When cells were doped with TiO_2_NPs, Young’s modulus values drastically increased to 372 ± 60 kPa for nucleus region and 549 ± 40 kPa for cytoplasmic region (Fig. 10b).

## Discussion

The spread of different kind of ENPs in several fields raises awareness about the importance to assess their potential toxicity in living organisms and the environments as well, taking into account their potential application in biomedical field [46–48]. In vitro and in vivo investigations are crucial to enrich the scientific knowledge and to release reliable clinical and epidemiological data [49]. The toxicity tests performed on different cells are considered the golden standard to assess the safety of NPs. However, few studies have investigated the interactions between NPs and cells from a biomechanical point of view. Cell mechanic is an important factor that influences many cellular pathways, including apoptosis, differentiation, migration, cancer metastasis, and wound healing [50]. In our work, we have addressed this point and related cell viability with the changes in mechanical properties of cells treated with different NPs. Firstly, we synthetized amorphous SiO_2_NPs and crystalline TiO_2_NPs with a size of c.a. 20 nm. NPs were stable in water and DMEM up to 96 h, even upon incubation with 10% and 20% of FBS. This was found to induce an increase in NPs size due to the formation of protein corona, in perfect agreement with the literature data. [51]. Since the entry route of NPs often occurs through inhalation and ingestion, we opted to investigate the potential effects on Caco-2 and A549 cells, which are representative models for the intestinal tract and airways mimicking oral and inhalation uptake [52]. As primary investigation, we quantified the cellular internalization of SiO_2_NPs and TiO_2_NPs by elemental analysis. The most effective uptake was observed in Caco-2 cells, especially upon treatment with TiO_2_NPs in a time-dependent manner. It has been reported that amorphous SiO_2_NPs, with a small size range of 15–20 nm, can bind the plasma membrane and then passively pass across the lipid bilayer to get access into the cells [53]. As demonstrated in A549 [54] and Caco-2 [55], in fact, small SiO_2_NPs can translocate in the cytoplasm with no apparent membrane encapsulation. The anatase crystalline form of TiO_2_NPs is the more chemically reactive [56] showing a faster absorption with respect to rutile, as previously reported [32]. However, the uptake mechanisms of Caco2 are still unclear, despite that some hypothesis have been formulated, some of these include metal ion release upon NPs degradation in the intestinal barrier lumen or/and direct uptake by endocytosis. [57]. In A549 cells, TiO_2_NPs were localized in cytoplasm and close the nucleus region [58]. We used WST-8 assay to assess the influence of different concentrations of SiO_2_NPs and TiO2NPs on cell viability. We have observed a general decrease of viability, especially in Caco-2, with TiO_2_NPs displaying the strongest toxicity. After assessing the viability, we monitored the extracellular release of the cytoplasmic enzyme LDH. We confirmed that the NPs induced an extensive membrane damage, which relates also to the increase of intracellular ROS levels, resulting in oxidative stress. In this context SOD, which acts as strong antioxidant against ROS [59], was significantly reduced most probably because of the unbalance of the redox repair systems. In addition, the oxidative stress increased the lipid peroxidation [60], as demonstrated by MDA measurements after NPs incubation. This is particularly evident in Caco-2 cells after TiO_2_NP exposure. It is worth mentioning that this effect can decrease membrane fluidity, which can further explain the observed higher entry levels of the TiO_2_NPs [61]. This was in significant accordance with the intracellular oxidative stress levels measured by SOD inhibition, as well as with the reactive oxygen species generation. After these assessments, we investigated the modulation of the cell cytoskeleton, as an increase of intracellular ROS could affect the F-actin organization [62]. The cytoskeleton is characterized by a set of filaments (actin microfilaments, microtubules, and intermediate filaments) organized in a network that affects the mechanical properties of cells, as well as their behavior [29]. In particular, actin filaments are crucial for cell mechanics, and any alterations may induce aberrations in cell morphology under sub-toxic conditions [63]. It has been demonstrated that actin was one of the most commonly bound protein by SiO_2_NPs and TiO_2_NPs in cellular extracts. This definitely suggests that the actin functions, as well as cell motility and organelles trafficking, can be strongly affected by the presence of these NPs [64, 65]. As a further proof, several in vivo studies have revealed the potential of NPs to induce alterations in the expression of genes related to the cytoskeleton [63]. In order to understand how NPs modulate the cytoskeleton, we performed qualitative and quantitative confocal analyses on Caco-2 and A549 cells, after SiO_2_NP and TIO_2_NP treatment. We focused on actin stress fibers and cortical actin because they allowed to maintain the physiological mechanical architecture of cells. As reported in Figs. 4 and 5, the treatment with NPs induced a significant reorganization of actin. This was more evident after 72 h of treatment with 45 μg/ml of NPs, and especially with the use of TiO_2_NPs. The adverse effects were stronger in intestinal cells, where we have observed the formation of protrusions and philopodia at the plasma membrane level, together with the disruption of tight junctions. Fluorescence coherency and fluorescence density have been used as quantitative parameters to assess alterations of actin distribution in the cytoskeleton. While coherency gives information about the organization of actin, density quantifies the levels of fluorescent actin. Caco-2 and A549 exposed to NPs showed a reduction of coherency compared to untreated cells, especially upon incubation with TiO_2_NPs. This was in good agreement with the qualitative confocal imaging analyses. The fluorescence density of actin was not altered by NP treatment in both the cell lines, even if untreated Caco-2 cells showed higher values with respect to untreated A549. These data could suggest a potential difference in the amount of actin, which is dependent on >the specific cell type. We also evaluated the nucleus-to-cytoplasm ratio as the relative area of the nucleus over the whole cell. We confirmed a reduction of values in A549 and an increase of the ratio in Caco-2 with respect to the control cells. This indicates changes in cell morphology after NP treatment: Caco-2 underwent an increase of nucleus area, whereas A549 became larger through cytoplasm extension. As a final point, we explored any potential change in cell elasticity upon NP treatment. Cell elasticity is commonly expressed by Young’s modulus (E), which is a ratio between the stress and the applied strain (with unit in Pascal) [66, 67]. Changes in cell elasticity due to cytoskeleton reorganization is often associated to disease progression [68], hence (E) can be a refined indicator of potential pathological states [67]. The deformability of cells was measured through indentation experiments by AFM [69]. Many studies showed the detrimental effects of NPs on the F-actin that induced an enhancement of cell elasticity. However, a clear relation between change in cell stiffness, actin rearrangement and cell viability has not been clearly established yet. Here, we have covered such topic and found that Caco-2 and A549 cells significantly change their (E) upon NP treatment, even though in two different ways. Caco-2 cells are softer as confirmed by the decreased Young’s modulus, which has been found to be a function of both the NPs type and the cell regions analyzed. In particular, TiO_2_NPs induced a general enhancement of elasticity, and this effect is more evident in the nuclear regions rather than the cytoplasmic one. On the other side, A549 displayed a remarkable increase of Young’s modulus after TiO_2_NP exposure in cytoplasm region, compared to control cells (594 ± 40 kPa versus 146 ± 26 kPa, respectively). These data indicated that TiO_2_NPs induce dose-dependent changes in force–deformation profiles in both cell lines, whereas SiO_2_NPs showed little effects. The decrease of Young’s Modulus, and consequently an increase of elasticity after NPs exposure, can potentially impact cell homeostasis and physiological pathways. The reorganization F-actin, together with a reduction of coherency, showed a strong modulation of mechanical cell properties. NPs have been demonstrated to make the nuclear region of Caco-2 cells softer than untreated cells. The increase of elasticity (corresponding to a reduction of Young’s modulus) is a critical factor in tumor progression, because it is an indicator of disruption of cell junctions, which promotes in turn cell migration and metastatization [70]. Therefore, the treatment with NPs on Caco-2 (and TiO_2_NP_S_ in particular) can potentially promote migration due to change of elastic properties and deformability of cells. Also, the larger and softer nucleus area can be associated to cancer progression [71].

## Conclusion

In this paper, we careful assessed the adverse effects of SiO_2_NPs and TiO_2_NPs on two different cell lines (Caco-2 and A549), mimicking the typical tissue that are exposed to NPs (ingestion and inhalation). SiO_2_NPs presented a low cytotoxicity in comparison with TiO_2_NPS. We demonstrated how the cellular exposure to high doses of NPs induced morphostructural changes in term of actin reorganization, coherency, density and nucleus/cytoplasm ratio, which were more evident upon TiO_2_NP treatment. Cell membrane deformability measurements showed different behavior in the two cells. In Caco-2, the cell elasticity increased after NP treatment, whereas A549 displayed an increase of stiffness. These results demonstrated that NPs induce modifications of cytoskeleton structures and, as consequence, a different Young’s Modulus values. Hence, the phenotype of cancer cells can turn into a more invasive profile, characterized by enhanced migration. On the other side, the increased stiffness observed in A549 might not promote the mobility of this specific cell indeed. We are sure that the analysis of cell mechanics upon NP exposure, combined with standard toxicological assays, will represent a golden standard to accurately assess the safety of NPs and to predict any potential possible diseases triggered by NPs.

## Abbreviations

A549: Human adenocarcinoma alveolar basal epithelial cells
AFM: Atomic force microscopy
ATP: Adenosine triphosphate
Caco-2: Human epithelial colorectal adenocarcinoma
CLSM: Confocal Laser Scanning Microscopy
DAPI: 4′,6′-Diamidino-2-phenylindole
DCF-DA: 2′,7′-Dichlorofluorescein diacetate
DLS: Dynamic light scattering
DMEM: Dulbecco’s modified Eagle’s medium
ENPs: Engineered nanoparticles
FBS: Fetal bovine serum
HCl/HNO3: Hydrochloric/nitric acid
ICP-AES: Inductively coupled plasma atomic emission spectroscopy
LDH: Lactate dehydrogenase
MDA: Malondialdehyde
NH4OH: Ammonium hydroxide
NPs: Nanoparticles
PBS: Phosphate Buffered Saline
ROIs: Regions of interest
ROS: Reactive Oxygen Species
SiO2NPs: Silicon dioxide nanoparticles
SOD: Superoxide dismutase
TBA: Thiobarbituric acid
TEM: Transmission electron microscopy
TEOS: Tetraethyl orthosilicate
TiO2NPS: Titanium dioxide nanoparticles
TTIP: Titanium (IV) isopropoxide
XRD: X-ray diffraction
